# Fundamentals of Force-Controlled Friction Riveting: Part I—Joint Formation and Heat Development

**DOI:** 10.3390/ma11112294

**Published:** 2018-11-15

**Authors:** Gonçalo Pina Cipriano, Lucian A. Blaga, Jorge F. dos Santos, Pedro Vilaça, Sergio T. Amancio-Filho

**Affiliations:** 1 Graz University of Technology, Institute of Materials Science, Joining and Forming, BMVIT Endowed Professorship for Aviation, 8010 Graz, Austria; goncalo.pinacipriano@tugraz.at; 2 Department of Mechanical Engineering, School of Engineering, Aalto University, FI-00076 Espoo, Finland; pedro.vilaca@aalto.fi; 3 Helmholtz-Zentrum Geesthacht, Center for Materials and Coastal Research, Institute of Materials Research, Materials Mechanics, Solid State Joining Processes, 21502 Geesthacht, Germany; lucian.blaga@hzg.de (L.A.B.); jorge.dos.santos@hzg.de (J.F.d.S.)

**Keywords:** friction, riveting, hybrid structures, joining, response surface

## Abstract

This work presents a systematic study on the correlations between process parameters and rivet plastic deformation, produced by force-controlled friction riveting. The 5 mm diameter AA2024 rivets were joined to 13 mm, nominal thickness, polyetherimide plates. A wide range of joint formations was obtained, reflecting the variation in total energy input (24–208 J) and process temperature (319–501 °C). The influence of the process parameters on joint formation was determined, using a central composite design and response surface methodology. Friction time displayed the highest contribution on both rivet penetration (61.9%) and anchoring depth (34.7%), and friction force on the maximum width of the deformed rivet tip (46.5%). Quadratic effects and two-way interactions were significant on rivet anchoring depth (29.8 and 20.8%, respectively). Bell-shaped rivet plastic deformation—high mechanical interlocking—results from moderate energy inputs (~100 J). These geometries are characterized by: rivet penetration depth of 7 to 9 mm; maximum width of the deformed rivet tip of 9 to 12 mm; and anchoring depth higher than 6 mm. This knowledge allows the production of optimized friction-riveted connections and a deeper understanding of the joining mechanisms, further discussed in Part II of this work.

## 1. Introduction

Nowadays, growing economic pressure and environmental concerns are pushing several industries to integrate alternative lightweight materials into their products [1]. As an example, for the transport industry, the usage of such materials constitutes an effective solution for reducing fuel consumption-associated costs and greenhouse gas emissions. This need for innovative designs must tackle several obstacles, such as joining different classes of materials without compromising the benefits from their individual usage.

The most commonly used methods to perform connections between dissimilar materials are mechanical fastening and adhesive bonding [2]. The latter consists on intermolecular forces created between the materials. Although it does not alter the mechanical properties of the materials, it has several drawbacks, for example the need for extensive surface preparation and long curing cycles. This type of connection is prone to degradation caused by environmental factors, such as moisture absorption and temperature [3]. Concerning the state-of-the-art of mechanical fastening, some limitations are related to the need for additional components, such as bolts and rivets, and to the pre-features necessary to accommodate them. These pre-features can constitute stress concentration points, affecting fatigue properties and corrosion resistance [2,4]. 

Welding can be also used for thermoplastic materials. A polymeric weld is generated from the localized melting of the material and/or the application of mechanical work [5,6]. The same cannot be said for thermosets, which do not soften due to their high-density cross-linked structure, degrading at high temperatures. These and other limitations associated with the aforementioned conventional processes, have led to the development of several alternative joining techniques. 

The present work focuses on one alternative joining technique, friction riveting (FricRiveting). The FricRiveting process was developed and patented by Helmholtz-Zentrum Geesthacht [7]. It was devised as a design solution to perform similar and dissimilar polymer and hybrid metal-polymer/composite overlapping joints. Since early studies by Amancio-Filho et al. [8], works have been carried out on assessing the influence of the process parameters on joint formation. By applying a design of experiments (DoE), Altmeyer et al. [9] demonstrated the feasibility of joining short carbon-fiber-reinforced polyether-ether-ketone (PEEK) with titanium grade 3. In their work, a geometric correlation was established to quantify the rivet tip deformation. This correlation was defined as a ratio between the amount by which the diameter increased at the tip of the rivet and its original dimension. Although this ratio gave an indication regarding mechanical performance of the joint produced, it did not take into consideration the shape of the deformed tip of the metallic rivet. Rodrigues et al. [10], also investigated joint formation on AA2024-T351 and polycarbonate joints, by determining a volumetric ratio for the deformed rivet tip and its correlation with joint tensile strength. This coefficient, earlier defined by Blaga et al. [11], establishes a simplified ratio between the volume of the plastically deformed rivet and the volume of polymer offering mechanical resistance to a rivet-pull-out action. Borba et al. [12] have investigated microstructural changes in the thermo-mechanically affected zone of the rivet, in Ti-6Al-4V/GFRP friction-riveted metallic-insert joints. The authors concluded that the process parameters influenced the local mechanical properties of the rivet, where microstructural changes were observed, with the occurrence of β to α phase transformation. In a recent work, Proença et al. [13] have demonstrated the feasibility of force-controlled friction riveting for short-fiber reinforced polyamide 6 and AA6056. By using a simple one-factor-at-a-time design of experiment (OFAT), the authors have investigated the individual influence of rotational speed, friction force, forging force, forging time, and rivet displacement (or displacement at friction), on the joint quasi-static mechanical performance for four different process conditions. Nonetheless, up to this point, there was no systematic investigation on the physics of the force-controlled friction riveting process. A deeper understanding of heat generation, joint energy efficiency, joint formation and mechanical performance is necessary. 

In this work, AA2024-T351 rivets and polyetherimide (PEI) metallic-insert joints were used to evaluate force-controlled friction riveting. These materials were selected due to both their mechanical properties and their frequent application in the aircraft industry. The current manuscript presents the first part of this extensive work, where the correlations between the process parameters and their influence on joint formation and energy development were investigated using a response surface methodology. A deep process understanding, over a wide range of rivet plastic deformation levels, is necessary to better control the joining mechanisms involved, and by doing so, the resulting joint strength. Considering a lean approach to the design of components and structures, it is of great importance that tailor-made joints can be produced. To accomplish this goal, it is necessary to establish models capable of yielding joining process parameters beforehand. Hence, based on statistical analysis of designed experiments, analytical models for the joint formation will be established. These models are meant to generate optimized process parameters capable of resulting in predetermined rivet deformations, which can yield a desired joint tensile strength, hence fulfilling the structural design specifications. The research methods implemented will enable an assessment of how the mechanical energy is used during the processing of the materials and influences the formation of the joint.

The hybrid joints produced and investigated in the present work were further analyzed and tested in the second part of the study (Part II). This second and final part of the work—published as a separate manuscript—focuses on the influence the joining process parameters have on joint mechanical performance and process energy efficiency, contributing to a deep and complete insight on the fundamentals of the force-controlled friction riveting process.

## 2. Force-Controlled Friction Riveting

**Figure 1:**
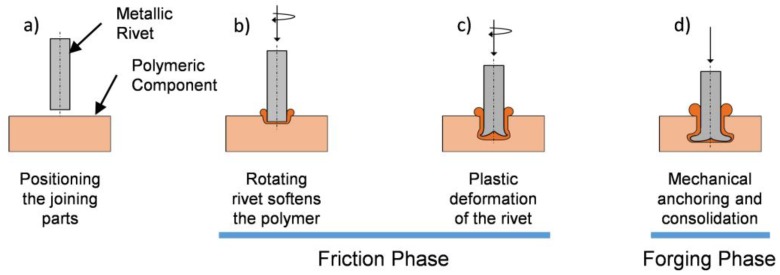
Schematics of the FricRiveting process using its basic configuration (metallic-insert joint geometry): (**a**) pre-joining configuration; (**b**) initial softened/molten polymer layer is formed; (**c**) plastic deformation starts; and (**d**) final deformation is achieved.

FricRiveting is a friction-based spot-joining alternative technology. This process can be performed using several set-up configurations [14] and be controlled by time [15], force [13], position, or by their combination through multiple-phases. A metallic rivet can be used with a plain featureless surface or can be profiled (e.g., threaded [8]). Figure 1 schematically represents the process basic configuration (devised for the manufacture of metallic-insert joints), when joining a non-profiled rivet to a single polymeric plate. The process is divided into three distinct stages: friction; forging and consolidation [8,11,15]. Initially, the rotating rivet is pressed against the polymeric plate. This generates heat by friction, promoting a local increase in temperature, which softens or melts the polymer (i.e., temperature above the glass transition temperature range for amorphous polymers, or the melting point for semi-crystalline polymers). The rivet is then inserted into the polymeric component while rotating. Due to the low thermal conductivity of the polymer, the heat generated is accumulated in the vicinity of the rivet tip. When sufficiently high temperatures are achieved, the tip is plasticized and starts to deform. The rotation is then reduced to a full stop. At this point, although plastic deformation has been achieved, a forging phase may also be applied before consolidation, with the axial downward force being increased, in order to further plastically deform the rivet, if desired. Finally, the tip of the rivet assumes either an anchor or bell-shaped axisymmetric geometry, consolidating under constant pressure. The process parameters are: rotational speed (RS); friction time (FT); friction force (FF); forging time (FoT); and forging force (FoF). These will be discussed in the following sections, but were fully addressed in previous publications [8,9,10].

For the present work, the process was carried out using a force-controlled and time-limited approach: the axial force being applied to the rivet is monitored and kept constant during the process, being the distinct phases limited by pre-defined time intervals. In this case, the insertion of the rivet into the polymeric plate is a process response, resulting from the evolution of the material conditions.

## 3. Materials and Methods

### 3.1. Base Materials

The materials used were AA2024-T351 (rivets) and Polyetherimide (PEI). The latter is a high-performance thermoplastic developed by Wirth et al. [16]. PEI is characterized by high mechanical strength, dimensional stability and flexural modulus. It also has good flame resistance, good chemical stability and an elevated softening point, with a glass transition temperature (T_g_) close to 215 °C [17,18]. By meeting specific flame resistance and low smoke evolution requirements, this material is used for both automotive and aircraft interior applications [19]. The PEI specimens were obtained from 13 mm nominal thickness extruded plates (Quadrant Engineering Plastic Products, Lenzburg, Switzerland). Specimens were cut into 70 mm × 70 mm format, for non-destructive X-ray analysis.

The metallic rivets used for this work were made of extruded AA2024-T351, with a length of 60 mm and a diameter of 5 mm. This is a solution heat-treated and cold-worked aluminum alloy naturally aged to a substantially stable temper condition (T3). The chemical composition of this alloy is presented in Table 1. The alloy is characterized by medium-to-high tensile strength (450 MPa) and it is widely used for aircraft primary structures, on fuselage and mechanical connections. Further details on the mechanical and physical properties of this alloy were addressed in the second part of this work. 

### 3.2. Joining Procedure

The joints performed for this study were produced using a FricRiveting lab-scale joining equipment (RNA, H. Loitz-Robotik, Hamburg, Germany). The equipment has a maximum rotational speed of 21,000 rpm and 24 kN of axial force. The force and torque measurements recorded by the sensors are used for controlling the process and provide an estimation of the mechanical energy input. For the present work, the friction time (FT) parameter varied from 1.4 up to 2.2 s and the forging time (FoT) from 0.5 up to 2.5 s. The rotational speed (RS) ranged from 17,000 up to 21,000 rpm, while the forces from 1500 up to 3500 N for friction (FF) and from 3300 up to 5700 N for forging (FoF). These parameter ranges were intentionally set so to promote a diversified range of resulting joint formation geometries and energy inputs.

### 3.3. Non-Destructive Joint Analysis

**Figure 2:**
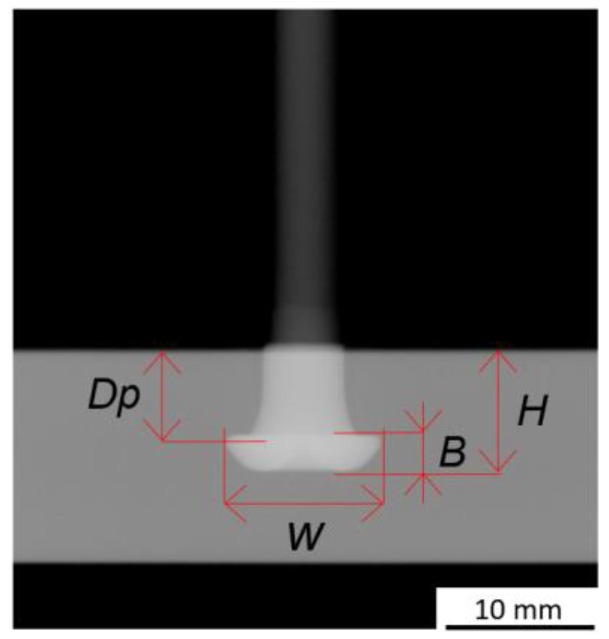
Example of X-ray tomography measurements for the joint formation analysis.

The joint formation of the specimens produced for mechanical testing, was evaluated by X-ray tomography. This analysis allowed the overall rivet projected geometry to be assessed, providing same-joint results for both joint formation and global mechanical performance (the latter discussed in Part II). Figure 2 exemplifies the tomographic measurements (Seifert Isovolt 320/13, Russia) performed in accordance with DIN EN ISO 17636-1, with a tube current of 5.4 mA and an 80 kV voltage. The focal spot was 1.5 mm × 1.5 mm at an 800 mm focus-to-film distance. The dimensions evaluated were: rivet penetration depth (H); maximum width of the deformed rivet tip (W); the height of the deformed rivet tip (B); and the depth until maximum width, or anchoring depth (Dp). The latter is introduced in the present work, as an improvement to the current approach on estimating rivet anchoring performance. This dimension depends on both radial deformation and penetration of the rivet tip into the polymer. The new joint formation assessment arose from the need to characterize the wide range of rivet plastically deformed geometries obtained, with the process variant and parameters being used. These measurements also serve as a basis to establish correlations with the mechanical performance in Part II.

### 3.4. Energy Input

In friction-based joining processes, the heat generated can be estimated by considering the mechanical energy input applied, both for processes involving metallic materials [21] and thermoplastics [22]. For the current work, the following equation was used to estimate this energy input [23,24],

(1) $$E_{M}=E_{f}+E_{d}=\int M.\omega .\mathrm{dt}+\int F.\vartheta .\mathrm{dt}\;(J),$$

where the first term is related to the frictional energy (E_f_) resulting from applied torque (M) and rotational speed (ω). The second term estimates the deformational energy (E_d_), resulting from the axial force (F) and the deformation rate (ϑ). The estimation of these energies, allows a correlation between the energy used and the resulting joint formation. With this data, it is possible to assess parameter combinations that result in more energy-efficient joints (i.e., joints resulting in more favorable plastically deformed rivet tip and yielding better mechanical properties, with less energy consumption). The torque was integrated over time (i.e., the area under the frictional torque curve), which when multiplied by the constant rotational speed gave the frictional component of the energy. The deformational energy was determined by multiplying the force being applied by the displacement over the whole process duration.

### 3.5. Temperature Measurement

**Figure 3:**
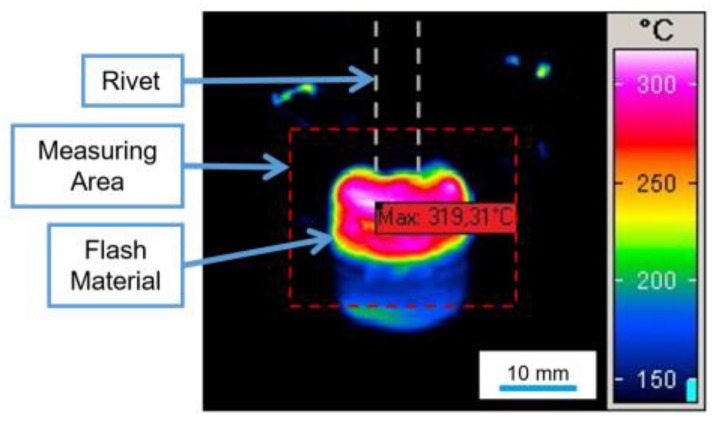
Example of infrared tomography measurements for the expelled flash material.

The process temperature was measured by infrared thermometry on the expelled polymeric flash material. A thermographic camera (High-End Camera Series ImageIR, Infratech GmbH, Dresden, Germany) was used with a calibration set for temperatures ranging from 150 up to 700 °C. The distance between the measuring area and the center of the lens was 60 cm, at an incidence angle of approximately 18°. Figure 3 shows a frame from the flash material temperature measurement during the process as an example.

### 3.6. Design of Experiments and Statistical Analysis

The parameter sets used to perform the joints for this work were established via a second order design of experiments. The aim was to define parameter sets that would fit a response surface to the experimental output studied [25,26]. The statistical input factors, joining parameters, were: FF; FT; RS; FoF; and FoT. The evaluated process responses for joint formation—i.e., the plastic deformation underwent by the rivet tip—were: H; W; and Dp.

The test matrix was generated by a central composite design (CCD), which integrates a factorial design, and both a set of center and one of axial points. For the factorial part, a fractional factorial design of five parameters with two levels (2^5−1^) was chosen. The value of α—the distance between the axial and the center points—was chosen as to give properties of rotatability and orthogonality to the design [26,27]. 

Response surface models, based on second order polynomial functions, were determined for the investigated responses. The statistical significance of all the linear, quadratic and two-way interaction model terms was evaluated at each iteration step by a stepwise backward elimination procedure. The least significant term was removed before the subsequent step and the model was revaluated. A polynomial regression equation was determined for each response, with an updated predictive capability when compared to the original full-quadratic model. Explanatory and predictability capabilities of the statistical models obtained were also evaluated. The response surfaces generated, for the statistically significant process parameter interactions, allow a deeper understanding into the effects these have on the studied response.

The parameter combinations set by the chosen DoE are shown in Table 2 in non-randomized order. The parameter window provides an understanding of the energy range necessary to achieve certain levels of deformation on the rivet tip and the resulting mechanical properties of the joints.

## 4. Results and Discussion

### 4.1. Joint Formation

The process-related plastic deformation, endured by the metallic rivet, was analyzed by non-destructive X-ray tomography, as described in Section 3.3. Table 3 shows the results of the measurements performed to assess the overall geometry of the plastically deformed rivet tip.

**Figure 4:**
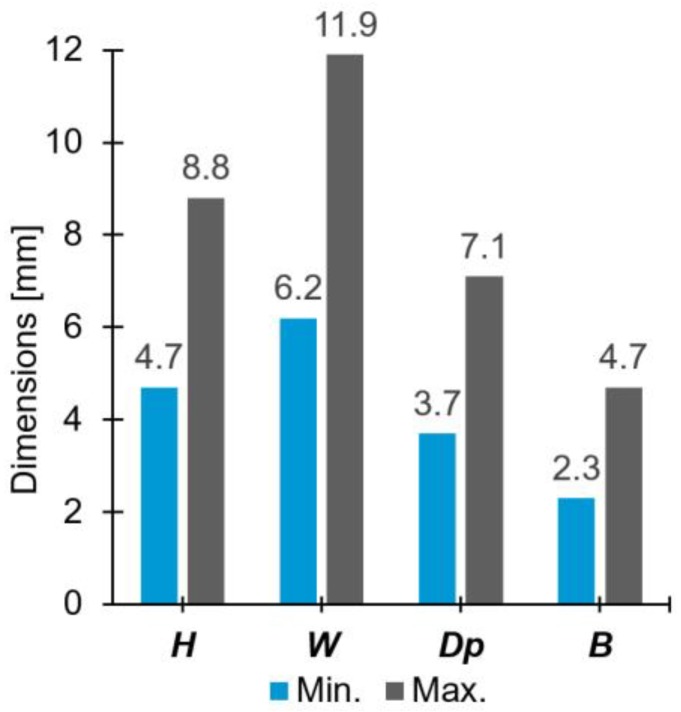
Maximum and minimum values of the rivet dimensions within the joint formation evaluation.

The parameter matrix yielded a wide range of rivet tip geometrical variations, as was intended. Figure 4 clearly presents the variation of the rivet dimensions.

The penetration achieved by the rivet (H) varied 46.6% of its maximum. The smallest width of the deformed tip (W) was 6.2 mm, 52.1% of the maximum and a 24% increase from the original rivet diameter of 5 mm. The anchoring depth (Dp) varied 47.9% of its maximum value. Finally, the height of the deformed rivet (B) range was approximately equal to the minimum value. The sensitivity of the process to the joining parameter sets is considerably high, hence the selected joining parameter window was adequately chosen for a large variation of results. A similar trend was observed by Amancio et al. [14], for the same combination of materials, but for a time-controlled process variant.

### 4.2. Mechanical Energy Input

The mechanical energy applied to the materials was estimated using Equation (3). The individual energy contributions, frictional (E_f_), deformational (E_d_), and the resulting total energy input values (E_M_) are shown in Table 4.

**Figure 5:**
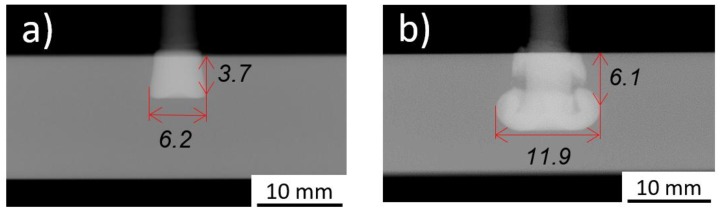
X-ray tomography of: (**a**) Condition 1 with lowest energy input, E_M_ = 24 J (RS = 18,000 rpm; FT = 1.6 s; FoT = 1 s; FF = 2000 N; FoF = 5100 N); and (**b**), Condition 16 with highest energy input, E_M_ = 208 J (RS = 20,000 rpm; FT = 2 s; FoT = 2 s; FF = 3000 N; FoF = 5100 N).

The range of parameters used for this work also yielded a large variation of energy contributions. In terms of total magnitude, the lowest value of the energy input was observed for Condition 1 (E_M_ = 24 J), and the highest with Condition 16, (E_M_ = 208 J). In Condition 1 (joint formation shown in Figure 5a), except for the forging force (FoF = 5100 N), the remaining joining parameters were at the second lowest level of their respective ranges (RS = 18,000 rpm; FT = 1.6 s; FF = 2000 N; and FoT = 1 s). Given the resulting joint formation, it was clear that the energy was not sufficient to promote significant plastic deformation of the rivet tip. Rodrigues et al. [10] observed similar results for friction riveting of policarbonate (PC) and AA2024-T351, reporting that lower RS, FT and FF (addressed as friction pressure in their work) lead also to lower process temperatures and a lower plastic deformation of the rivet tip. Condition 16 (joint formation shown in Figure 5b) was produced with a higher energy input, associated with higher joining parameters: 11% more rotational speed (RS = 20,000 rpm); 25% friction time (FT = 2 s); and 50% friction force (FF = 3000 N), than Condition 1. This is also in agreement with Rodrigues et al. [10], who observed an increase in H and W with these joining process parameters. In Condition 16, a rupture of part of the rivet tip outer edge was then separated from the main rivet body, remaining close to the surface of the polymer (indicated by arrows in Figure 5b). This type of defect was reported as a result of an unsteady rivet plastic deformation related to local oscillations in polymer viscosity due to excessive heat generation [14].

**Figure 6:**
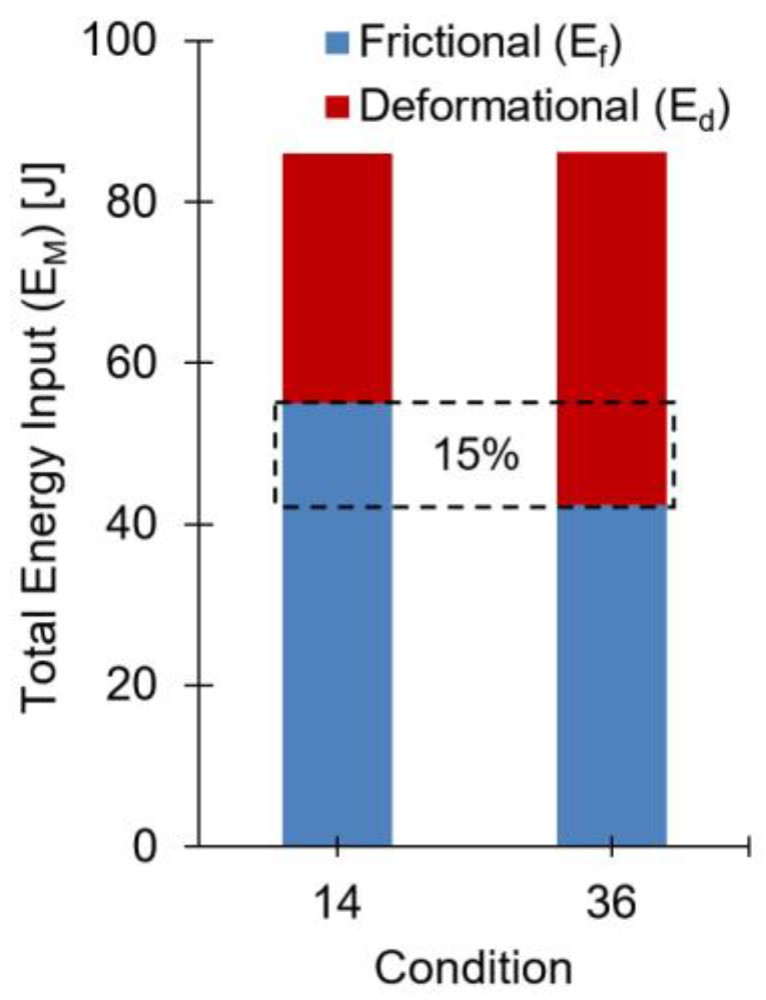
Energy input comparison between Conditions 14 and 36 with total energy input of 86 J.

Besides the total amount of energy applied during the process, the balance between the individual energy contributions was also found to be determinant to the joint formation. When analyzing Conditions 14 and 36, both having the same total energy input of 86 J, the influence from the energy balance on rivet deformation can be established. Figure 6 demonstrates the balance between the frictional (E_f_) and deformational (E_d_) contributions to the total energy input on these two conditions.

**Figure 7:**
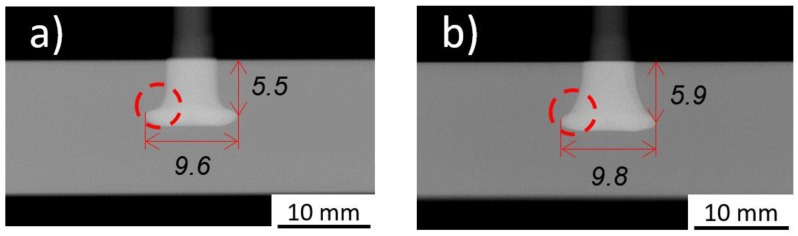
X-ray tomography of: (**a**), Condition 14 (RS = 20,000 rpm; FT = 1.6 s; FoT = 2 s; FF = 3000 N; FoF = 3900 N); and (**b**), Condition 36 (RS = 19,000 rpm; FT = 1.8 s; FoT = 1.5 s; FF = 2500 N; FoF = 5700 N).

The 15% of the total energy input that in Condition 14 originates from a frictional contribution, in Condition 36 the same 15% generated by deformational energy. This shift in the predominant source of energy promoted a considerable change in the resulting joint formations. Referring to the data presented in the previous section (Table 3), the measurements performed on these joints revealed higher dimensions for the deformed rivet tip of Condition 36. The highest difference in magnitude occurred for the B measurement, encircled in Figure 7, where Condition 36 had an increase of over 30% from the value measured for Condition 14.

The dashed-circle in Figure 7b, highlights a smoother, more gradual transition, from the original diameter to the maximum width of the deformed rivet tip. This can be considered a direct result from the difference in magnitude of the forging forces involved. The joint produced with Condition 14 was produced with a FoF of 3900 N, while the joint from Condition 36 with 5700 N. Apparently, this difference promoted higher compression in the rivet for the latter condition, which, by encountering sufficient resistance from the solid polymer in front, resulted in a larger volume of metallic material being forced to expand its diameter, at a point closer to the polymer exterior surface. 

The results indicate the importance of the energy balance on the deformation of the rivet. This was found to be another important factor, which, independently from the global energy, can greatly influence the plastically-deformed rivet geometry. As was reported in [11] and as will be shown in Part II, the shape of the deformed rivet tip plays an important role in dictating joint tensile mechanical performance. Therefore, one of the energy contributions may be preferable to the other, or even a balance might be desirable. An immediate example of this will be further discussed in the next section, where, E_f_ is decisive to maximize Dp.

### 4.3. Influence of the Process Parameters on Joint Formation

The process responses H, W and Dp were statistically investigated based on a response surface design, CCD, as described in Section 3.5. Statistical models will be further discussed for each response separately.

#### 4.3.1. Influence on the Rivet Penetration Depth, H

**Figure 8:**
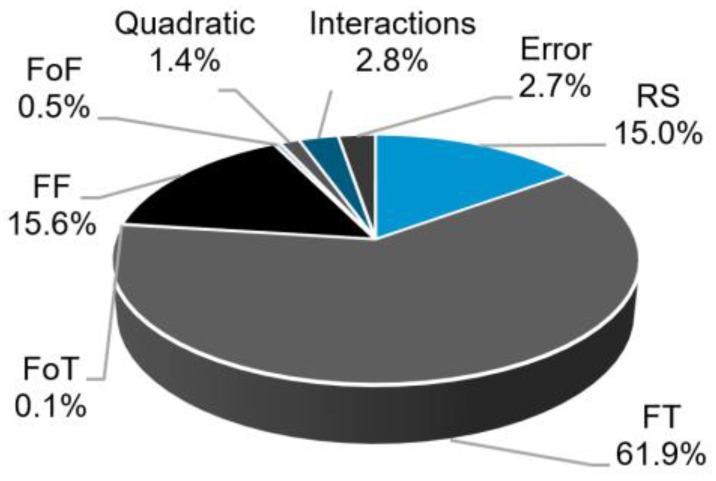
Contributions of the model factors to the response H.

The reduced statistical model for H was obtained following the procedure described in Section 3.5. Using analysis of variance, the terms considered significant for this study were those with a *p*-value inferior to 0.05 [25,26]. The RS, FT and FF were the most significant parameters, with *p*-values of zero. FoF and FoT had values of 0.041 and 0.435, respectively. Although FoT had a high *p*-value, for the FT*FoT and FoT*FoF interactions this value was 0.026 and 0.014, respectively. Therefore, it was kept in the reduced model on a principle of hierarchy. Likewise, the interaction FT*FoF was considered significant with *p* = 0.001. The relative contributions of the process factors to the H model are shown in Figure 8. The largest individual contributions to H come from FT (61.9%), RS (15.0%) and FF (15.6%). This is in accordance with the work reported by Amancio-Filho and dos Santos [28], where these joining parameters directly influence heat generation in the viscous polymer layer, allowing for a larger rivet penetration. Moreover, Equation 1 shows that the longer the joining time the higher will be the energy generated, explaining the largest contribution of FT. Finally, the two-way interactions and quadratic terms, although statistically significant, display only a small combined contribution (4.2%) to this response. Both FoF and FoT individual contributions were found to be marginal, inferior to the model total error.

Equation (2) presents the reduced model regression equation in CCD matrix coded values for H:

(2) $$\begin{array}{c} H=6.7264+0.4375\mathrm{RS}+0.8875\mathrm{FT}-0.0292\mathrm{FoT}+0.4458\mathrm{FF}-0.0792\mathrm{FoF}-0.0865\mathrm{FF}*\mathrm{FF}+0.0760\mathrm{FoF}\\ {}*\mathrm{FoF}+0.1062\mathrm{FT}*\mathrm{FoT}+0.1687\mathrm{FT}*\mathrm{FoF}+0.1188\mathrm{FoT}*\mathrm{FoF} \end{array}$$

**Figure 9:**
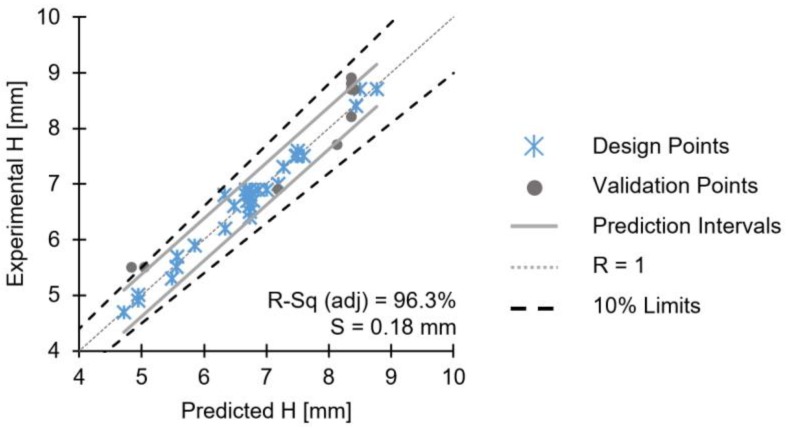
Validation diagrams for the reduced model of H.

A comparison between the predicted and the observed values of H is established in Figure 9, along with additional validation experiments. The dotted line follows a 1:1 correlation between the axes. The solid grey lines enclose the upper and lower prediction limits, within which the model can predict a single response observation [29].

The adjusted R-sq—the explanatory power of the model—was 96.3%, the standard error (S) 0.18 mm, and the predicted R-sq 93.8%. Apart from one point (predicted value of 6.4 mm/actual value of 6.8), all observations lie within the prediction limit interval. From the validation runs, for the conditions closer to the extreme H values, the experimentally obtained values are higher than the predicted ones. This may be related to a limitation in model prediction power when near the extremities of the joining parameter ranges. However, these validation points might be influenced by a usual 10% uncertainty associated with variability in polymer rheological properties such as molecular weight distribution (MWD) between different grades and batches. Considering that average molecular weight and MWD affect molten viscosity [30], polymerization-related variations in temperature, time and reactor type may be enough to alter polymer base material rheological behavior [31,32] during FricRiveting. 

**Figure 10:**
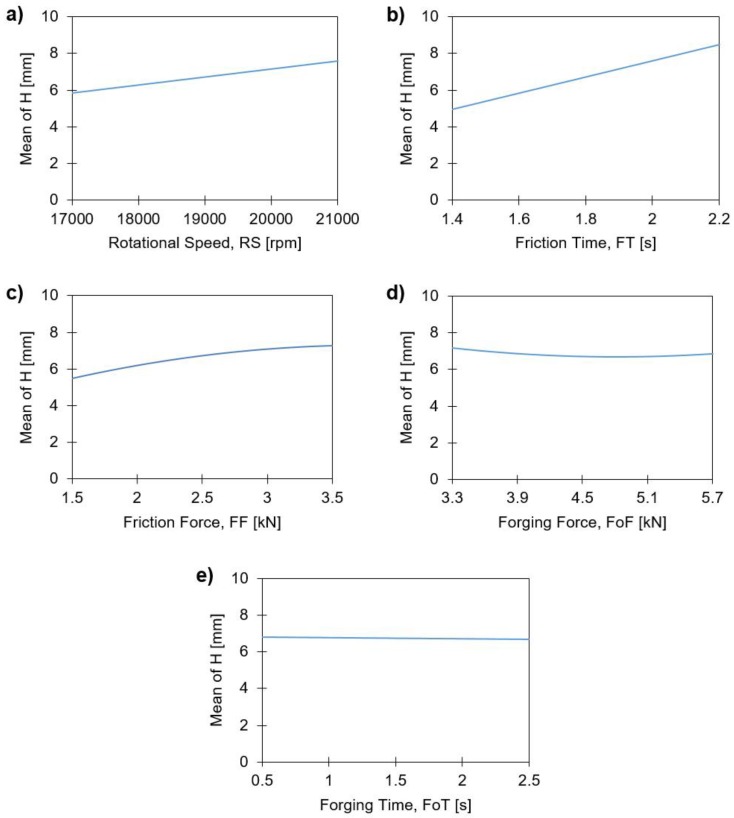
Main effects plots of the rivet penetration depth, H, with: (**a**) rotational speed; (**b**) friction time; (**c**) friction force; (**d**) forging force; and (**e**) forging time.

The influence process parameters have on rivet penetration depth, H, is shown in Figure 10, by the main effects plots.

**Figure 11:**
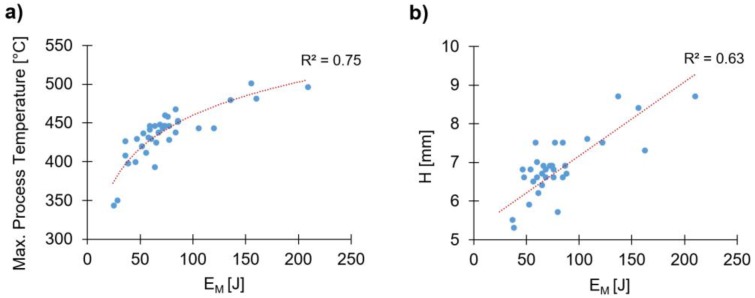
Total energy input, E_M_, correlations with: (**a**) maximum process temperature; and (**b**) rivet penetration depth, H.

For the parameter ranges being studied, H increases with the individual values of RS and FT (Figure 10a,b). These display a relatively linear correlation with H, also reported by Altmeyer et al. [9] for Ti alloy/short-fiber reinforced polyether ether ketone joints. In the cases of FF (Figure 10c) and FoF (Figure 10d), a slight curvature can be observed, whereby for the former, this effect is more prominent, suggesting a higher order influence. The curvature of FF can be explained by taking into account that the increase of this parameter leads to an increase of E_M_. One may assume, that a larger FF will promote a non-linear decrease in the polymer viscosity. This effect can be coupled on one hand with the increase in process temperature with E_M_ (Figure 11a), and on the other hand, with the thixotropic behavior of PEI [33]. Larger FF values will increase the shear rate, consequently promoting a decrease in polymer viscosity. A similar explanation can be extended to RS and FT, as these joining parameters also generate higher energy input (Equation (1)). This relative tendency can be seen in Figure 11b, with the correlation between H and E_M_. Therefore, the higher the RS, FT and FF the larger will be the volume of softened polymer ahead of the rivet, offering less resistance to rivet insertion. This behavior is in agreement with previous works [8,9]. Finally, the FoT curve (Figure 10e) shows relatively no influence on the magnitude of H.

**Figure 12:**
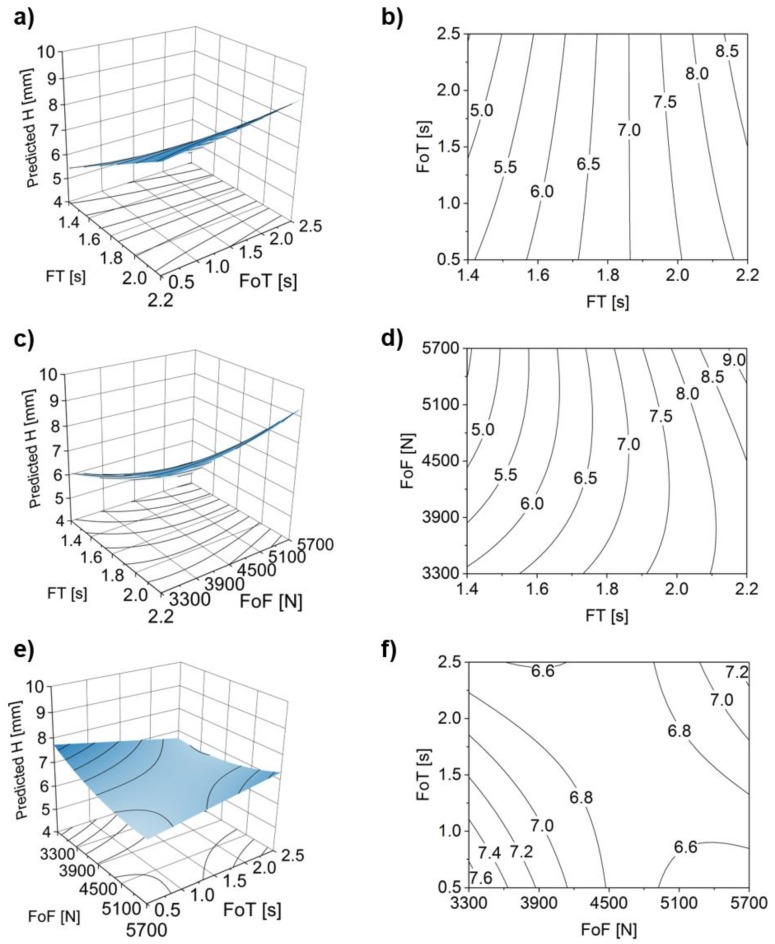
Two-way parameter interactions with rivet penetration depth (H) as response. Surface and contour plots for: (**a**,**b**) friction time (FT) and forging time (FoT); (**c**,**d**) FT and forging force (FoF); and (**e**,**f**) FoT and FoF.

Figure 12 shows the corresponding response surface and contour plots, supporting the analysis of statistically significant two-way interactions (FT*FoT, FT*FoF and FoF*FoT) considered in the model.

When analyzing the FT*FoT interaction for FoT >1.5 s (Figure 12a,b), both minimum and maximum values of H (i.e., H < 5 and H > 8.5 mm) are achieved by respectively applying minimum and maximum values of FT. The rivet penetration increases linearly with the increase of FT as discussed in Figure 10. The FT*FoF interaction surface plot (Figure 12c) shows a pronounced curvature. Although the same tendency of H increasing with the FT is observed, there is a considerable gradient at the lower half of the FoF parameter range (3300 N ≤ FoF ≤ 4500 N). Contrary to what could be expected, an increase in FoF for the lower values of FT (1.4 s ≤ FT ≤ 1.6 s), leads to a decrease of H for this parameter range (Figure 12c,d). A possible explanation could be that (Figure 10b) at lower FT the amount of molten polymer ahead of the rivet will be smaller, as less energy is being applied to the system. For FoF, the observed influence over the range is considered small, as it was seen for the respective main effect plot (Figure 10d). Figure 12e and f show that the FoT*FoF interaction is the one producing the smallest variation in magnitude for H, across this parameter range. This trend is in agreement with the results in Figure 10e, where FoT has no relative influence in H. Therefore, one may conclude that FoT is not contributing significantly to H in the selected joining parameter range. Finally, one may derive from the surface plots that FT is the parameter with the greater influence on the two-way interactions.

#### 4.3.2. Influence on the Maximum Width of the Deformed Rivet Tip, W

**Figure 13:**
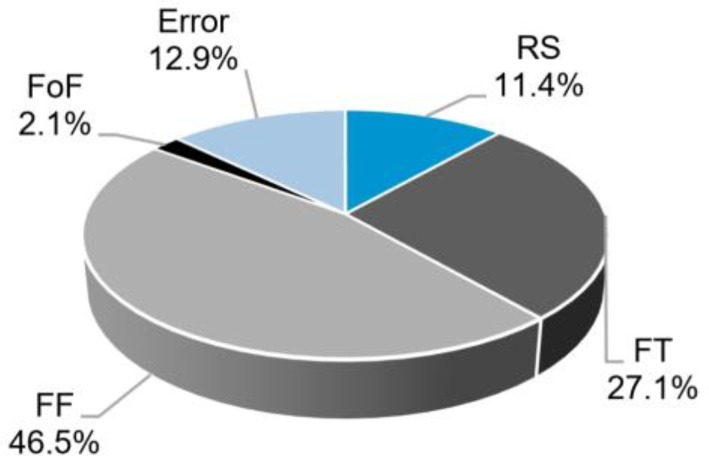
Contributions of the model factors to the response W.

The initial factors with *p*-values > 0.05, were all eliminated by the stepwise backward elimination methodology. The RS, FT, FF (*p*-value of zero) and FoF (*p*-value of 0.032) were found to be significant for W. Figure 13 shows the individual contributions of the model factors to W.

The largest contribution for W is generated by FF (46.5%). This can be explained by the fact that this process parameter promotes higher energy input and rivet deformation, hence the increase in W. The FT (27.1%) and RS (11.5%) also have relevant contribution on this response. These observations are in accordance with [9,10]. 

The regression equation for this model is:

(3) W=8.7472+0.596RS+0.912FT+1.196FF+0.254FoF

**Figure 14:**
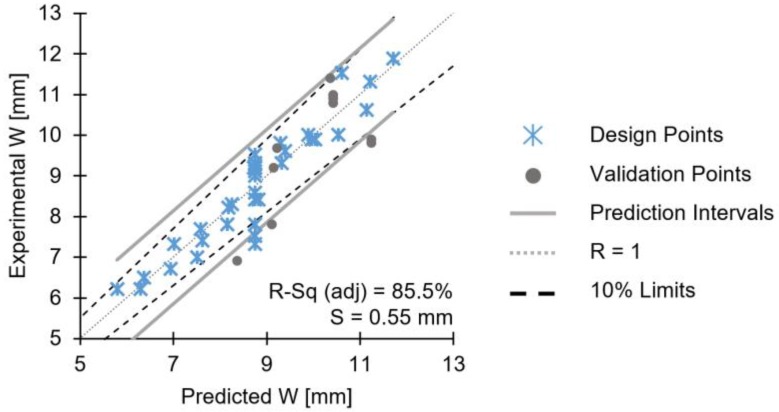
Validation diagrams for the reduced model of W.

Figure 14 shows the validation plot for the W model, where experimental values are plotted along with model predicted values.

The adjusted R-sq of the model is 85.5%, the standard error, S, is 0.55 mm and the predicted R-sq 84.4%. With the exception of two outliers outside the prediction intervals (experimental W values of 7.3 and 7.5 mm), the W model has a good explanatory power (i.e., large R-sq [25]).

The validation points for W, display good correlation between the experimental and the predicted values (i.e., large predicted R-sq), although some of the former fall on or just below the prediction interval. This may be correlated with the 10% uncertainty limits related to intrinsic batch-associated properties deviation. No interactions were found to be statistically significant, hence, they were not considered for the reduced model. Therefore, response surface graphs for two-way interactions were not investigated.

**Figure 15:**
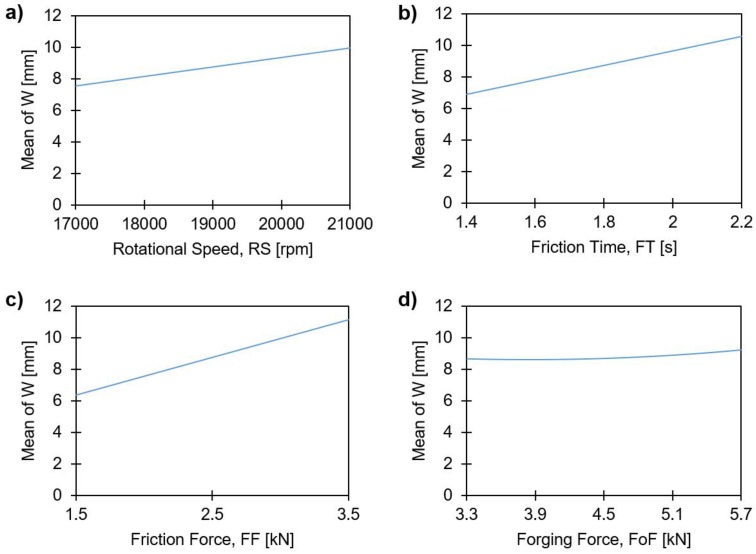
Main effects plots of the maximum width of the deformed rivet, W, with: (**a**) rotational speed; (**b**) friction time; (**c**) friction force; and (**d**) forging force.

Figure 15 presents the main effects plots for the statistically significant joining parameters, influencing the maximum deformed width of the rivet tip (W).

For the joining parameters RS (Figure 15a), FT (Figure 15b) and FF (Figure 15c), a linear correlation with W was found, whereby the higher these joining parameters, the larger will be the W values. FoF shows a minor curvature (Figure 15d) with what can be considered a very small variation of W (8.6 to 9.3 mm) given the studied force range. Taking into account that FoF has a rather irrelevant influence on H (refer to Figure 10d), this is an indication that the forging phase may exert only a minor influence on the formation of the rivet anchoring zone for the investigated joining parameter ranges. This has been previously suggested by Proença et al. [13] for force-controlled FricRiveting. They observed that for joints produced with lower energy inputs, a larger FoF would lead to a larger W, while for high energy input conditions, such as in the current study, FoF had lesser influence. The authors explained this phenomenon through the higher process temperatures measured for higher energy input conditions, which increase rivet tip plasticizing. They assumed that this was a result of rivet plastic deformation taking place during the heating phase. This is an indication that a forging phase with FoF higher than FF is not necessarily needed to deform the rivet tip, if it is possible to achieve enough heat generation through the appropriate selection of RS, FT and FF. 

**Figure 16:**
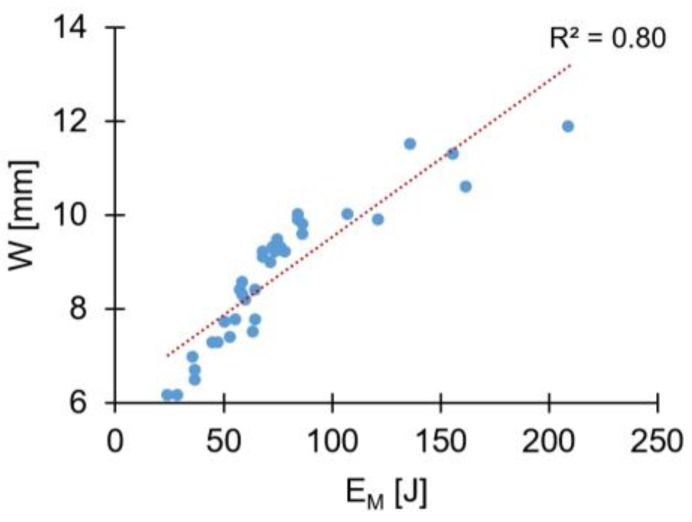
Correlation between maximum width of the deformed rivet tip (W), and the total mechanical energy input (E_M_).

The linear proportionality between RS, FF, FT, and W can be better understood by addressing the correlation of these parameters with the mechanical energy input (Equation (1)). The graph in Figure 16 demonstrates the tendency of W to increase with the mechanical energy input. Past studies [8] on this material combination, have shown that the higher the energy input the higher will be the heat generation. This could also be seen in the present study, as proven by the correlation between the process temperature and energy input (Figure 11a). Therefore, the level of plasticizing of the rivet tip will also increase, leading to larger radial plastic deformation and higher W. 

#### 4.3.3. Influence on the Anchoring Depth, Dp

The anchoring depth is being introduced in this work as a tool to better correlate the shape of the deformed rivet tip with the joint mechanical performance. The Dp will be used in Part II of this work to allow a more accurate assessment on the volume of material, above the deformed rivet tip, offering resistance to mechanical solicitation. As previously mentioned in the introduction, a correlation between this volume and the resistance to pullout forces has been established in literature.

A reduced statistical model with only significant terms was generated with RS, FT, FF*FF and FT*FF (*p*-value of 0.000). The model also included the quadric terms FT*FT (*p*-value of 0.004) and FoF*FoF (*p*-value of 0.047), as well as two-way interaction terms (the *p*-values were 0.019 for RS*FF and 0.028 for FT*FoF). Even though the *p*-values for the first order terms of FF (*p*-value of 0.410) and FoF (*p*-value of 0.264) are outside the interval of confidence (95%) set for the analysis of variance (ANOVA), they were considered in the reduced model due to hierarchy, as they are part of the two-way interactions.

**Figure 17:**
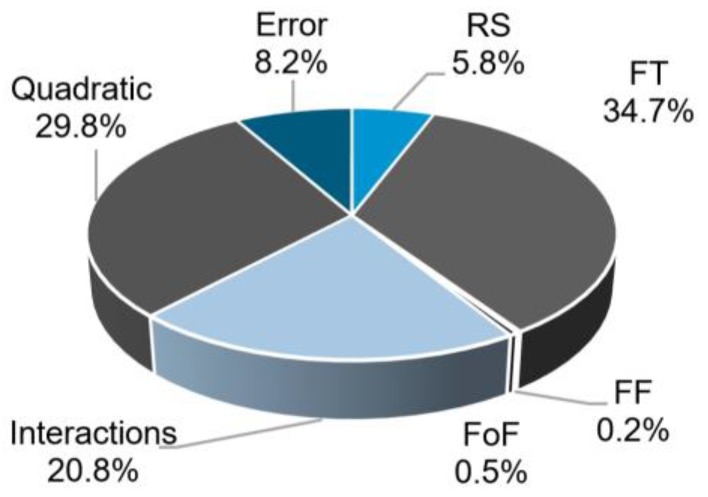
Contributions of the model factors to the response Dp.

Figure 17 shows the relative contributions of the model factors to Dp.

Similar to the case of H, the contribution of FT to Dp is the largest. Nonetheless, in contrast to H, the contributions from the quadratic terms and two-way interactions are the second (29.8%) and third (20.8%) largest ones, respectively. As previously mentioned in Section 3.3, Dp is a measure that depends both on the penetration of the rivet and on the amount of plastic deformation that its tip undergoes. Thus, the mechanisms in play are more complex than for the previous responses. This will be discussed further in this section.

The model regression equation for Dp is:

(4) $$\begin{array}{c} \mathrm{Dp}=5.9243+0.2292\mathrm{RS}+0.5625\mathrm{FT}-0.0458\mathrm{FF}-0.0625\mathrm{FoF}-0.1510\mathrm{FT}*\mathrm{FT}-0.4135\mathrm{FF}*\mathrm{FF}+\\ 0.0990\mathrm{FoF}*\mathrm{FoF}-0.1688\mathrm{RS}*\mathrm{FF}-0.4812\mathrm{FT}*\mathrm{FF}+0.1563\mathrm{FT}*\mathrm{FoF}, \end{array}$$

**Figure 18:**
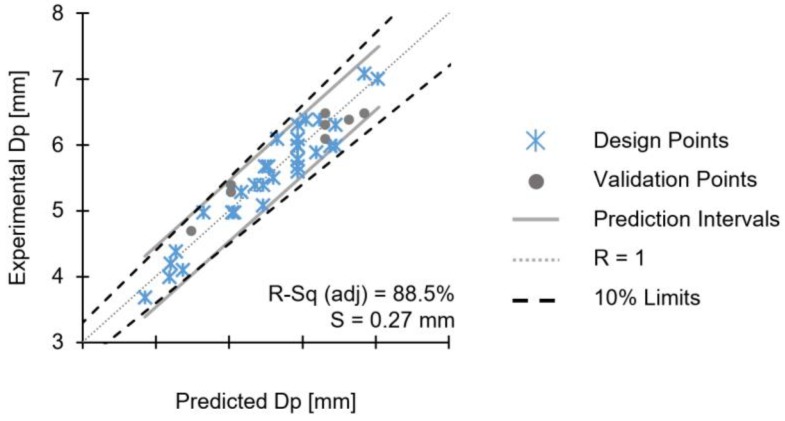
Validation diagrams for the reduced model of Dp.

The experimental Dp values measured are shown comparatively to the model predictions, in Figure 18.

The model has an adjusted R-sq of 88.5%, the predicted R-sq is 77% and a standard error, S, of 0.27 mm. The graph shows that all the design points fall within the model prediction interval, considered a good correlation with the experimental data. For Dp, all the validation runs performed fall within the prediction intervals. Therefore, the model can be used to explain the correlations with the joining parameters and predict Dp.

**Figure 19:**
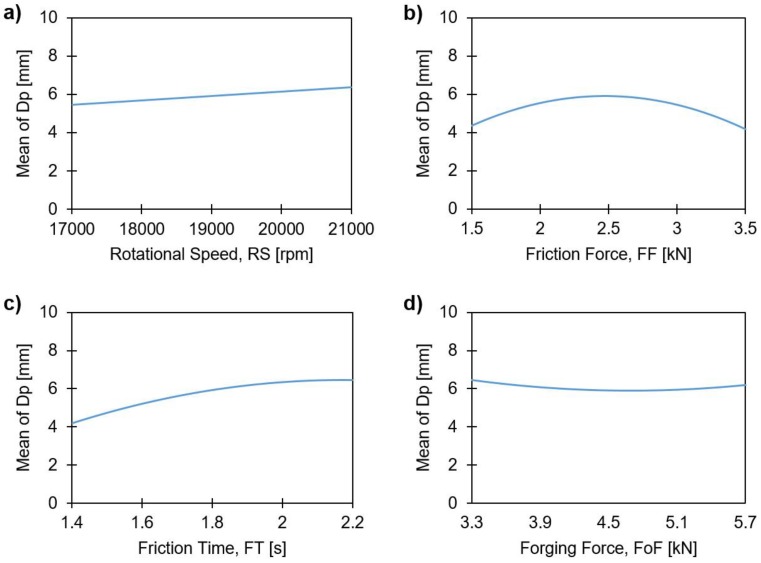
Main effects plots of the anchoring depth, Dp, with: (**a**) rotational speed; (**b**) friction time; (**c**) friction force; and (**d**) forging force.

The main effects plots showing the influence of the process parameters on Dp are shown in Figure 19.

**Figure 20:**
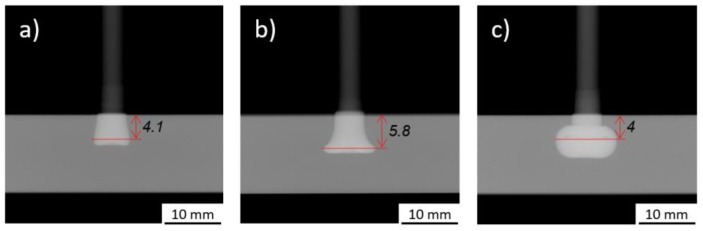
Friction force direct effect on Dp: (**a**) Condition 33 (RS = 19,000 rpm; FT = 1.8 s; FoT = 1.5 s; FF = 1500 N; FoF = 4500 N); (**b**) Condition 25 (RS = 19,000 rpm; FT = 1.8 s; FoT = 1.5 s; FF = 2500 N; FoF = 4500 N); and (**c**) Condition 34 (RS = 19,000 rpm; FT = 1.8 s; FoT = 1.5 s; FF = 3500 N; FoF = 4500 N).

As can be seen, only RS (Figure 19a) displays a linear correlation, in this case increasing, with Dp. FF (Figure 19b), FT (Figure 19c) and FoF (Figure 19b) plots indicate an influence from higher-order terms, as there is no linear correlation in these cases. In the case of FF, data shows a relative symmetry over the parameter range, having a maximum Dp of 6 mm close to the median values of friction force. At both minimum and maximum FF, Dp decreases to values close to 4 mm. This behavior may be explained by the rivet deformation regime during the process. A higher level of FF leads to a higher amount of plastic deformation at the rivet tip, as reported for W (Figure 15c). The more plasticized the metallic material being pressed, the more pronounced is the anchor-shape geometric effect of the rivet tip. Figure 20 demonstrates the effect of increasing FF from minimum (1500 N) until maximum (3500 N) values with the remnant process parameters kept constant. As Dp is the depth measured until the widest rivet tip deformation, this point occurs closer to the original upper surface of the polymer, as the plastic deformation of the rivet tip excessively increases. Given that the anchor-like deformed tip begins to curve upon itself, e.g., Figure 20c, in contrast to a bell-shape deformation seen in Figure 20b.

For the FoF, we observed similar behavior that observed for H (Figure 12d) and W (Figure 15d).

**Figure 21:**
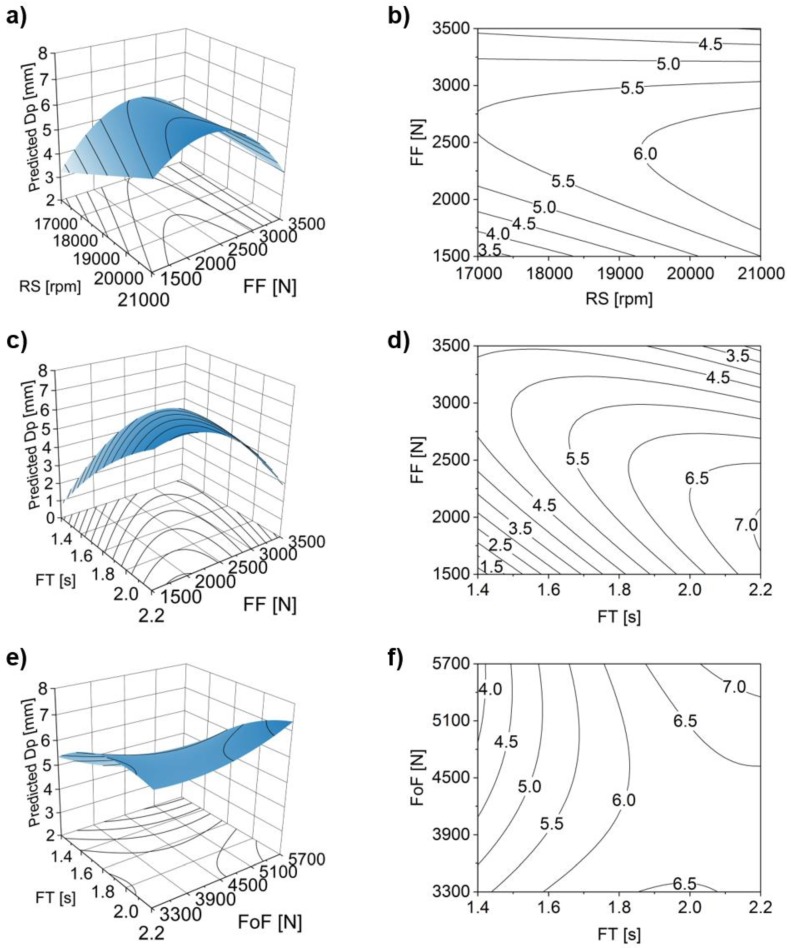
Two-way parameter interactions with anchoring depth, Dp. Surface and contour plots for: (**a**) rotational speed (RS) and (**b**) friction force (FF); (**c**) friction time (FT) and (**d**) friction force (FF); and forging time (FoT), (**e**), and forging force (FoF) (**f**).

The two-way interactions between parameters, for Dp, are illustrated in Figure 21.

The behavior observed from the main effects plot of FF (Figure 19b), seems to be amplified by the RS*FF interaction. Figure 21a,b suggest that when FF is set close to its maximum, the increase in RS will result in smaller Dp values. This can be related, as mentioned in Section 4.3.1, to resultant high-energy input. The higher the combined RS*FF, the larger the plastic deformation of the rivet tip, resulting in an increase of W. Besides the widening of the rivet tip, changes in shape will also take place, as addressed in Figure 20, creating long curved upon themselves anchor-like arms in the rivet tip (Figure 20c). The overall minimum Dp that is found at lower levels of FF and RS (i.e., at very low energy inputs), is associated with a very small penetration of the rivet. In these cases, no considerable deformation (i.e., lower W values, Figure 5a) is expected. 

The FT*FF surface and contour plots (Figure 21c,d) display the widest range of predicted Dp values, where FT appears to have a greater influence on Dp than FF. Also, an increase of FT should be coupled with a decrease in FF to maximize Dp. Similarly, as has already been discussed for Figure 20, increasing FF at high levels of energy input, contributes to an effect of over-deformation, seen by the sharp decrease of Dp at highest levels of FF and FT.

In the case of FT*FoF (Figure 21e,f), up to a certain level of FT (around 1.8 s) the increase of FoF towards maximum values becomes counterproductive for the increase of Dp. High FoF is only positive for Dp when FT is also close to maximum. This apparently complex behavior may be explained by two phenomena. Values of FoF around 4500 N seem to be enough to cause the rivet to further penetrate the polymer (Figure 12c,d) but not enough to promote an over-deformation, seen at higher values with the decrease of Dp (Figure 20c). At both maximum levels of FT and FoF, the Dp also achieves maximum values. This is due to the fact, that the gain in the rivet penetration outweighs the negative effect of over-deformation. 

#### 4.3.4. Summary of the Findings

A correlation between the joint mechanical performance and the volume of polymeric material above the deformed rivet tip has been established in previous studies [8]. To complement the existing knowledge, a fine control of the process-resulting rivet plastic deformation was now established. 

The interaction plots in Figure 21 for Dp, suggest that FF must be chosen carefully, as it is the parameter which promotes the greatest variations in the two-way interactions. These interactions emphasize the importance of the energy input balance on the joint formation (Section 4.2), and geometry of the deformed rivet tip (Figure 5b). Moreover, it is clear that a simple variation of the global energy is not sufficient to tailor Dp (Figure 20). In order to prevent the occurrence of rivet over-deformation, premature increase of W, and the resulting decrease of Dp (e.g., Figure 20c), the frictional contribution to the energy input must be controlled. The effect the FF increase had on energy input, from 68 J (Condition 25, Figure 20b) to 159 J (Condition 34, Figure 20c), resulted in a plastically deformed anchor-shaped rivet tip for the latter, in contrast to the bell-shape rivet tip for the former.

As verified in the main effects plots (Figure 10, Figure 15 and Figure 19) and the investigated two-way interaction surfaces (Figure 12 and Figure 21), for the three responses—H, W and Dp—the FoF and FoT parameters themselves either did not promote significant variation among these responses, or their contributions were very small. Therefore, these two joining parameters may be kept constant for process optimization purposes. Furthermore, the RS was found to have a positive linearly increasing effect on all three responses (Figure 10a, Figure 15a and Figure 19a). In this way, RS should be maximized to promote optimized joint formation, i.e., bell-shape rivet tip. This rivet shape results in improved joint mechanical performance, as will be discussed in Part II.

**Figure 22:**
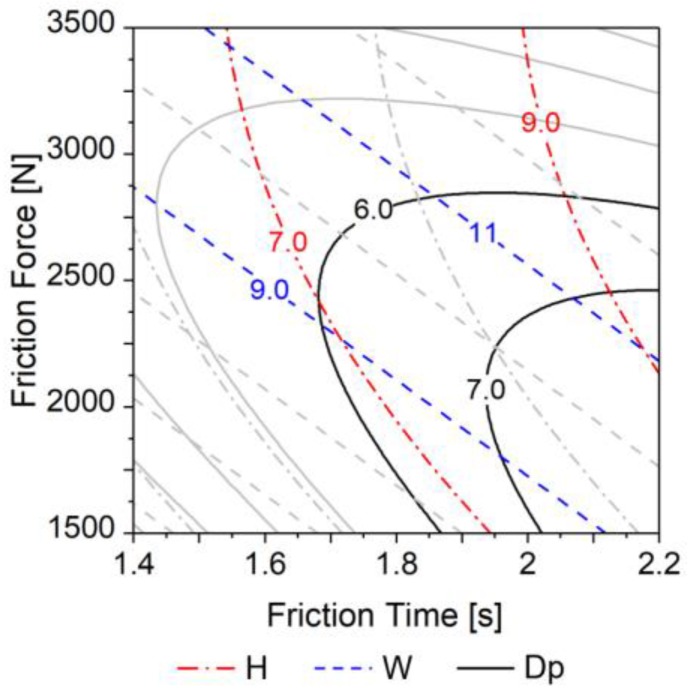
Influence of most relevant joining parameters FF and FT on joint formation (H, W and Dp). Values in millimeters. Regions of interest delimited by colored lines. RS is set at 21,000 rpm, FoT at 1.5 s and FoF at 4500 N.

To illustrate the dependence of investigated H, W and Dp geometric responses on FF and FT (compiled into Figure 22), FoF and FoT were set in their mid-range values (FoF = 4500 N; FoT = 1.5 s) and RS to its maximum value (RS = 21,000 rpm).

This graph can provide the user with a process overview, allowing for a tailored selection of the process parameters according to the requirements and constraints of a specific application. For instance, to maximize the polymeric resistive volume of material above the rivet tip—a requirement for improved joint mechanical performance—it is not enough to only increase H and W, using upper-range values of both FT (2 ≤ FT ≤ 2.2 s) and FF (3000 ≤ FF ≤ 3500 N). This would sharply decrease Dp (i.e., from over 6 mm to below 3 mm at maximum values of FT and FF), resulting in an over-deformed rivet tip (Figure 5b), an undesired feature. Hence, the use of process parameters which yield such over-deformation may be considered as energetically inefficient. As will be addressed in more detail in Paper II, having Dp values close to those of H maximizes the resistive polymer volume above the rivet tip. This desired feature could be achieved with the process parameter window where the following conditions in the contour plot of Figure 22 intercept: Dp higher than 6 mm; H between 7 and 9 mm; and W between 9 and 12 mm. Part II will focus on comprehensive discussions regarding the correlation between the resultant rivet tip geometry and joint mechanical performance.

## 5. Conclusions

Joint formation mechanisms of the force-controlled friction riveting process variant were systematically investigated, for the first time, in this work. A wide range of parameters was set using a central composite design of experiments. The statistical significances and influences of the process parameters on the resulting joint were determined using statistical analysis of variance and response surface methodology. The investigated joining parameter matrix yielded great variation of the measured rivet geometric responses characterizing the plastic deformation of the AA2024-T351 rivet tip inside the PEI plates. The energy input ranged between 24 and 208 J. Higher energy led to lower anchoring depth, reflecting excessive rivet plastic deformation. Process temperature also varied considerably across the parameter range (319–501 °C), resulting from the energy input. The frictional energy contribution proved decisive to the control of the overall rivet plastic deformation and process temperature, with moderate energy input levels (~100 J) preferable, avoiding excessive rivet deformation, which promotes decreasing joint mechanical performance.

The plastic deformation at the rivet tip was measured by rivet penetration depth, H (4.7–8.8 mm), maximum width of the deformed rivet tip, W (6.2–11.9 mm), and anchoring depth, Dp (3.7–7.1 mm). These responses were found to be influenced by the process parameters in different ways, with the magnitude and nature of such influence varying considerably. Rivet penetration depth was largely dependent on friction time (i.e., 61.9% of the total statistical contribution). For the maximum width of the deformed rivet tip, friction force and friction time contributed the most, with 46.5% and 27.1%, respectively. This response demonstrated a relatively linear increase in magnitude with increasing energy input. The anchoring depth displayed the most complex behavior of all three measurements. It was considerably influenced by quadratic (29.8%) and two-way interaction terms (20.8%). Similarly, in the case of rivet penetration depth, the anchoring depth also had its greater contribution deriving from friction time (34.7%).

The friction force parameter was found to have great influence on the geometry and final shape of the rivet tip. To avoid counterproductive over-deformation, both friction force and friction time should be set in a manner which does not promote excessive energy input. This excess of energy results in an undesirably low anchoring depth, reducing the resistive polymer volume above the rivet tip, responsible for offering resistance to a pull-out mechanical solicitation applied to the joint. The knowledge obtained with this work, on geometrical characterization and predictive models for joint formation, could allow for a tailor-made approach in the production of force-controlled friction-riveted joints. 

The second and final part of this work will be published as a separate manuscript in this journal (Part II), focusing on the mechanical performance and energy efficiency of friction-riveted joints, based on the knowledge on joint formation and energy input gained in the present Part I.

## Figures and Tables

**Table 1:** Typical nominal chemical composition of AA2024-T351 [20].

Element	Al	Cr	Cu	Fe	Mg	Mn	Si	Ti	Zn
**Weight (wt%)**	90.7–94.7	≤0.10	3.8–4.9	≤0.50	1.2–1.8	0.3–0.9	≤0.50	≤0.15	≤0.25

**Table 2:** Process parameter sets used for this study.

Condition	Process Parameters					Condition	Process Parameters				
	RS (rpm)	FT (s)	FoT (s)	FF (N)	FoF (N)		RS (rpm)	FT (s)	FoT (s)	FF (N)	FoF (N)
**1**	18,000	1.6	1	2000	5100	**19**	19,000	1.8	1.5	2500	4500
**2**	20,000	1.6	1	2000	3900	**20**	19,000	1.8	1.5	2500	4500
**3**	18,000	2	1	2000	3900	**21**	19,000	1.8	1.5	2500	4500
**4**	20,000	2	1	2000	5100	**22**	19,000	1.8	1.5	2500	4500
**5**	18,000	1.6	2	2000	3900	**23**	19,000	1.8	1.5	2500	4500
**6**	20,000	1.6	2	2000	5100	**24**	19,000	1.8	1.5	2500	4500
**7**	18,000	2	2	2000	5100	**25**	19,000	1.8	1.5	2500	4500
**8**	20,000	2	2	2000	3900	**26**	19,000	1.8	1.5	2500	4500
**9**	18,000	1.6	1	3000	3900	**27**	17,000	1.8	1.5	2500	4500
**10**	20,000	1.6	1	3000	5100	**28**	21,000	1.8	1.5	2500	4500
**11**	18,000	2	1	3000	5100	**29**	19,000	1.4	1.5	2500	4500
**12**	20,000	2	1	3000	3900	**30**	19,000	2.2	1.5	2500	4500
**13**	18,000	1.6	2	3000	5100	**31**	19,000	1.8	0.5	2500	4500
**14**	20,000	1.6	2	3000	3900	**32**	19,000	1.8	2.5	2500	4500
**15**	18,000	2	2	3000	3900	**33**	19,000	1.8	1.5	1500	4500
**16**	20,000	2	2	3000	5100	**34**	19,000	1.8	1.5	3500	4500
**17**	19,000	1.8	1.5	2500	4500	**35**	19,000	1.8	1.5	2500	3300
**18**	19,000	1.8	1.5	2500	4500	**36**	19,000	1.8	1.5	2500	5700

**Table 3:** Geometry variations in the plastically deformed rivet tip.

Condition	Joint Formation Measurements				Condition	Joint Formation Measurements			
	H (mm)	Dp (mm)	B (mm)	W (mm)		H (mm)	Dp (mm)	B (mm)	W (mm)
**1**	4.7	3.7	2.8	6.2	**19**	6.8	6.1	2.6	9.3
**2**	6.8	5.0	3.8	7.3	**20**	6.9	6.1	3.2	9.2
**3**	6.8	6.4	2.8	7.4	**21**	6.5	6.0	3.0	7.8
**4**	7.5	7.0	4.7	9.3	**22**	6.6	6.0	3.5	8.6
**5**	4.9	4.4	2.3	6.2	**23**	6.6	6.0	2.7	7.3
**6**	5.5	5.0	3.2	7.0	**24**	6.8	6.0	3.2	9.1
**7**	6.9	6.4	4.4	7.8	**25**	6.6	5.8	3.8	9.2
**8**	7.5	7.1	3.2	8.4	**26**	6.9	6.3	3.3	9.0
**9**	6.2	5.7	2.5	8.2	**27**	5.9	5.1	3.0	7.7
**10**	6.6	5.3	3.7	10.0	**28**	7.5	6.0	3.6	9.9
**11**	7.6	5.7	3.8	10.0	**29**	4.9	4.2	2.4	6.7
**12**	8.4	5.4	4.2	11.3	**30**	8.7	6.0	3.3	11.5
**13**	5.7	5.0	3.0	9.2	**31**	6.7	5.8	3.0	8.4
**14**	6.7	5.5	2.9	9.6	**32**	6.9	6.0	3.4	9.3
**15**	7.5	5.4	3.1	9.9	**33**	5.3	4.1	3.0	6.5
**16**	8.7	6.1	4.7	11.9	**34**	7.3	4.0	5.4	10.6
**17**	6.4	5.6	3.1	7.5	**35**	7.0	6.3	2.6	8.3
**18**	6.6	5.7	3.4	9.5	**36**	6.9	5.9	4.2	9.8

**Table 4:** Energy input determined for the investigated set of joining conditions.

Condition	Energy Input			Condition	Energy Input			Condition	Energy Input		
	E_f_ (J)	E_d_ (J)	E_M_ (J)		E_f_ (J)	E_d_ (J)	E_M_ (J)		E_f_ (J)	E_d_ (J)	E_M_ (J)
**1**	10	14	24	**13**	45	33	78	**25**	36	32	68
**2**	26	20	46	**14**	55	31	86	**26**	40	31	71
**3**	33	20	53	**15**	82	39	120	**27**	28	23	51
**4**	39	38	77	**16**	122	86	208	**28**	46	37	83
**5**	14	14	29	**17**	39	25	63	**29**	17	19	36
**6**	16	20	36	**18**	42	34	76	**30**	84	52	136
**7**	35	30	65	**19**	42	32	74	**31**	36	28	64
**8**	41	16	57	**20**	42	31	73	**32**	40	32	73
**9**	36	24	60	**21**	29	27	56	**33**	21	17	38
**10**	40	43	83	**22**	30	28	59	**34**	88	72	159
**11**	63	43	106	**23**	24	23	47	**35**	34	24	59
**12**	90	66	155	**24**	37	30	67	**36**	42	44	86
